# Factors Affecting Graft Survival among Patients Receiving Kidneys from Live Donors: A Single-Center Experience

**DOI:** 10.1155/2013/912413

**Published:** 2013-06-26

**Authors:** Mohamed A. Ghoneim, Mohamed A. Bakr, Ayman F. Refaie, Ahmed I. Akl, Ahmed A. Shokeir, Ahmed B. Shehab El-Dein, Hesham M. Ammar, Amani M. Ismail, Hussein A. Sheashaa, Mahmoud A. El-Baz

**Affiliations:** ^1^Department of Urology, The Urology & Nephrology Center, Mansoura, Egypt; ^2^Division of Nephrology, The Urology & Nephrology Center, Mansoura, Egypt; ^3^Division of Immunology, The Urology & Nephrology Center, Mansoura, Egypt; ^4^Division of Pathology, The Urology & Nephrology Center, Mansoura, Egypt

## Abstract

*Introduction*. The aim of this report is to study the graft and patient survival in a large cohort of recipients with an analysis of factors that may affect the final outcomes. *Methods*. Between March 1976 and March 2008, 1967 consecutive live-donor renal transplants were carried out. Various variables that may have an impact on patients and/or graft survival were studied in two steps. Initially, a univariate analysis was carried out. Thereafter, significant variables were embedded in a stepwise regression analysis. *Results*. The overall graft survival was 86.7% and 65.5%, at 5 and 10 years, respectively. The projected half-life for grafts was 17.5 years and for patients was 22 years. Five factors had an independent negative impact on graft survival: donor's age, genetic considerations, the type of primary immunosuppression, number of acute rejection episodes, and total steroid dose during the first 3 months after transplantation. *Conclusions*. Despite refinements in tissue matching techniques and improvements in immunosuppression protocols, an important proportion of grafts is still lost following living donor kidney transplantation, presumably due to chronic allograft nephropathy.

## 1. Introduction

In March 1976, the first renal transplantation in Egypt was carried out at the Department of Urology, University of Mansoura. A mother donated one of her kidneys to her daughter who was suffering from end-stage renal disease secondary to chronic pyelonephritis. Armed only with azathioprine and corticosteroids, the operative procedure and the functional outcome were very successful. A typical example of beginner's luck. Following a very slow start, the number of procedures increased gradually until it has currently reached a rate exceeding 80 cases every year. Herein, our long-term results are reported with an analysis of factors which can have an impact on patients and graft survival.

## 2. Patients and Methods

### 2.1. Patients

 Between March 1976 and March 2008, 1967 consecutive living donor renal transplants were carried out at the Urology & Nephrology Center, Mansoura University, Egypt. There were 1620 related and 347 unrelated donors. For recipients, our exclusion criteria included sensitization with positive lymphocytotoxic cross match and donor specific antibodies, recent malignancy, addiction, psychiatric disorders, type I diabetes mellitus, and significant extrarenal organs failure (pulmonary, hepatic, and cardiac). Absolute contraindications for donation included active infections, diabetes, even minimal renal function impairment, arterial hypertension, and serological positivity for HBV or HCV. Over the years, different immunosuppression regimens were utilized with or without an induction treatment. Azathioprine-based therapy was initially employed. Later on, a cyclosporine-based protocol was adopted [1]. Subsequently, triple immunosuppression regimens were utilized [2] Currently, steroid and/or calcineurin inhibitor-free protocols are the subject of several clinical trials [3, 4].

Approval of our Institutional Review Board (IRB) Committee was obtained.

### 2.2. Follow-Up

Patients were closely and regularly followed up for evaluation of renal function, the onset of surgical or medical complications, and side effects of immunosuppression. The median observation period was 7.47 years (range: 0–31.15 years).

### 2.3. Statistical Analysis

Actuarial survival was estimated by the Kaplan-Meier method. The log derivation of the percent survival was utilized for construction of graphs to predict the half-life of grafts and/or patients. Univariate analysis was carried out to correlate the graft survival to various preoperative, operative, and postoperative variables. Differences in survival were determined by the log-rank test. A *P* value of ≤0.05 was considered significant. For the determination of those factors who had an independent impact on graft survival, a Cox proportional hazard analysis was utilized. The variables included in this analysis were only those which were significant with the univariate study.

## 3. Results

**Figure 1:**
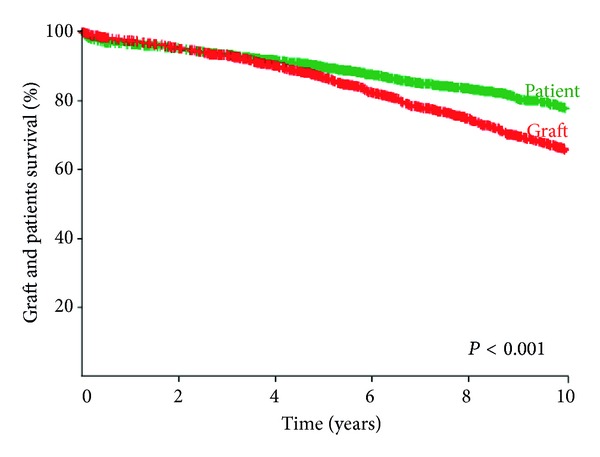
Actuarial patient and graft survival. Patient survival was 89.7 ± 0.7% and 77.8 ± 1.2% years at 5 and 10 years, respectively. Graft survival was 86.7 ± 0.8% at 5 years and dropped to 65.5 ± 1.3% at 10 years.

**Figure 2:**
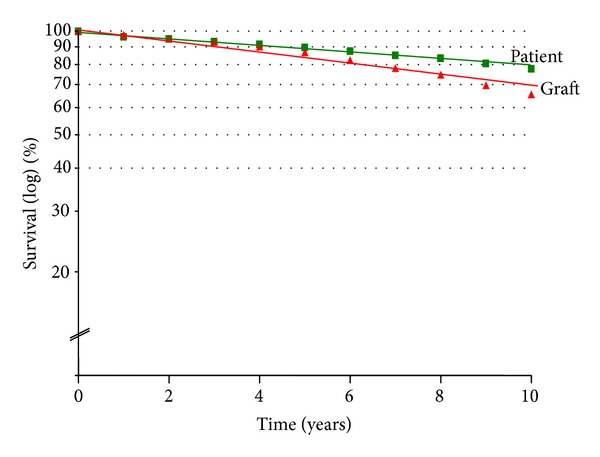
Log derivation of percentage of survival. The projected half-life for patients was 22 years and for grafts was 17.5 years.

The overall actuarial graft survival was 86.7% and 65.5% at 5 and 10 years, respectively. The corresponding patient survival was 89.7% and 77.8% (Figure 1). The graft survival was essentially stable throughout the first 5 years. A negative and steady decline was observed thereafter. The projected half-life for grafts was 17.5 years and for recipients was 22 years (Figure 2). 

Of the demographic variables, 3 had a significant negative impact on graft survival: donor's sex, donor's age, and recipient's age (Table 1(a)). Our data suggest that recipients who did not receive a blood transfusion had a better outcome (Table 1(b)). The number of class I and/or class II mismatches had a significant negative impact on graft survival. When both classes were pooled together, 2 observations could be made: the greater the number of mismatches the poorer was the result. Furthermore, there was a very clear separation in the prognostic outcome if the total number of mismatches was 2 or less versus 3 or more. It was also noted that HLA-identical siblings had the best short- and long-term graft and patient survival (Table 1(c)). The nature of the original kidney disease, if it was identified, had a marginal negative impact on graft survival. Other current or past medical disorders did not have an influence (Table 1(d)). 

**Figure 3:**
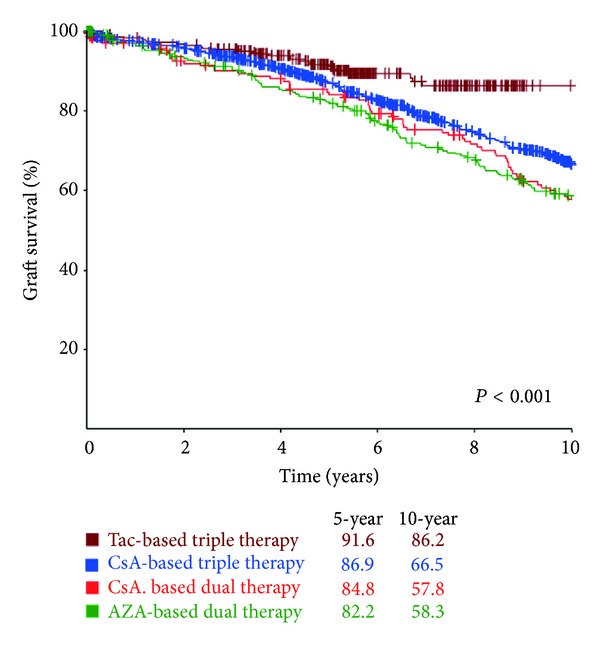
Graft survival relative to primary immunosuppressive regimen. Univariate analysis indicated that Tac-based triple therapy provided the best outcome.

Of the technical variables, 2 had a significant effect on graft survival: the time to diuresis and the total ischemia time (Table 2). Four post-transplant factors had a significant impact on graft survival: the primary immunosuppression (Figure 3), the number of acute rejection episodes, the total dose of steroid during the first 3 months, and post-transplant hypertension (Table 3).

 Of all the factors which had a significant impact on graft survival by univariate analysis, only 5 sustained their significance when the step-wise regression analysis was carried out (Table 4). In this analysis, evidence was provided that donor's age, genetic considerations, primary immunosuppression, number of acute rejection episodes, and total steroid dose during the first 3 months acted as independent variables which had a significant influence on graft survival.

## 4. Discussion

Over recent years, there has been a global increase in the number of live-donor kidney transplants in view of a severe shortage in the availability of deceased donor organs [5, 6]. The results of live-donor transplantation are generally superior to those obtained from deceased donors [7]. This can be explained, at least partially, by the short ischemia time. Factors inherent to the transplanted organ itself can also play an important role. Live donors are subject to rigorous predonation assessment. Furthermore, they are not exposed to major cardiovascular instability, sepsis, or nephrotoxic agents that may occur during hospitalization before declaration of brain death [8]. Our study relies only on an analysis of results of living donation. The overall graft survival was 86.7% and 65.5% at 5 and 10 years, respectively, with a projected half-life of 17.5 years. This compares favorably with recently published data [7, 9]. Out of the 1967 donors, 347 were unrelated (16.7%). The probability of graft survival among related and unrelated donors was essentially similar. This is consistent with previously published reports [10]. It is of interest to note that the use of unrelated donors led to a higher graft survival than related ones in certain conditions, namely, type I DM, focal glomerulosclerosis and polycystic kidney disease [11].

In our report, factors that can possibly have an influence on graft and patient survival were evaluated by univariate as well as by multivariate analysis. Out of all the studied variables, only 5 had an independent impact on the graft survival: the donor's age, the genetic considerations, the type of primary immunosuppression, the number of acute rejection episodes, and the total dose of steroids during the first 3 months after transplantation. It is noteworthy that the postoperative urologic complications in this series did not have a significant impact on patient and graft survival. The nature of such complications and their impact on patient and graft survival had been fully addressed in a previous report [12].

There is some controversy regarding the impact of the donor's age on the outcome of renal transplantation from living donors. In our series, there was a negative impact on graft survival of kidneys obtained from donors above the age of 40 years. Similar observations were also reported by several investigators [9, 13, 14]. Reduced pre-donation GFR, higher blood pressure, and total cholesterol levels, usually associated with older age, were suggested as a possible explanation. However, patient and graft outcomes from older living kidney donors were reported as similar to those from younger donors despite lower GFR [15].

In an earlier report, it was observed that when the combined number of HLA-A, -B and -DR mismatches was considered, the influence of the degree of HLA incompatibility was significant [16]. In the current study, such an impact was not seen which is in agreement with a recently published report [9]. The increased number of patients who received a calcineurin inhibitor can provide an explanation. Nevertheless, the best outcomes, by far, were obtained from HLA identical siblings. Results of graft survival of kidneys obtained from 1-haplotype and 2-haplotype mismatched pairs were not different. This is in agreement with data from the collaborative transplant study (CTS) [17]. 

Evidence was provided that a tacrolimus-based triple therapy provided the best probability of graft survival at ten years. A similar finding was also reported by the CTS [16]. In their study, a combination of tacrolimus/azathioprine (Tac/Aza) provided a better result than that of tacrolimus/mycophenolate mofetil (Tac/MMF). A possible explanation is that while Tac/MMF can provide a more potent immunosuppression, Tac/Aza is associated with a reduced risk of infectious complications [18]. 

Our data demonstrate that the incidence and number of acute rejection episodes encountered during the first 3 months after transplantation had a significant negative impact on graft survival. This is also reflected in the inverse relationship between graft survival and the total dose of corticosteroids during the same time period. A long list of predisposing factors for acute rejection can be compiled. Nevertheless, there are no reliable method(s) to predict whether a given patient will have a rejection. On this basis, it was suggested that the routine utilization of an induction regimen could be of benefit. To this end, the use of an interleukin-2 receptor antibodies is attractive because of their potential role in the prevention of rejection with almost negligible side effects [19, 20]. In a prospective randomized controlled study with an extended follow-up, Sheashaa and associates reported that basiliximab induction reduces the incidence of acute rejection significantly. However, a noticeable effect on the long-term renal transplant outcome was not seen [21]. 

The independent and significant negative influence of the cumulative dose of corticosteroids received during the first 3 months after transplantation is not a surprise. Their multiple adverse effects on the patient's blood pressure, lipid profile, and glucose tolerance are well documented [22, 23]. Hence, studies were carried out to explore the feasibility of corticosteroid withdrawal or minimization. These had provided evidence of the clinical benefits from early or late steroid withdrawals without an untoward effect on graft survival [24–26]. In a meta-analysis by Knight and Morris, they reported that steroid avoidance after renal transplantation increases the risk of acute rejection but decreases the cardiovascular risks. They concluded that such protocols are justified with current immunosuppressive protocols in low-risk recipients [27]. A similar conclusion was reported by our group when a steroid-avoidance immunosuppression regimen was utilized for low-risk live-donor transplant recipients [3].

Despite the significant improvements in graft survival achieved by the introduction of calcineurin inhibitors, the nephrotoxicity of these agents had raised concern [28, 29]. In a longitudinal histological study, it was demonstrated that long-term calcineurin inhibitor nephrotoxicity is common and characterized by increasing small vessel-narrowing and progressive ischemic glomerulosclerosis [30]. This nephrotoxicity can account for the fact that reduction of early episodes of acute rejection did not result in a corresponding improvement in the long-term outcome. In our study, the excellent results obtained at 5 years after transplantation, followed by a steady decline in graft survival. This can be, at least partially, due to calcineurin inhibitor-induced nephrotoxicity. Several studies explored the possibility of calcineurin-inhibitor sparing and/or steroid avoidance immunosuppressive regimens. The best maintenance immunosuppressive regimen with calcineurin inhibitor and/or steroid sparing is a work in progress and awaits a longer follow-up. One strategy employs the utilization of a powerful agent for induction to be followed by withdrawal of the calcineurin inhibitor after a predetermined time. We have conducted a pilot study to determine the feasibility of steroid and calcineurin inhibitor-free regimen following live-donor kidney transplantation [31]. Alemtuzumab was used for induction. Tacrolimus was given for the first 2 months and replaced by sirolimus thereafter. Out of 41 recipients, 31 are enjoying steroid-free regimen of whom 21 patients are calcineurin inhibtor-free as well. This regimen proved to be effective with good patient acceptance and excellent patient and graft survival. The reported follow-up period of 28.3 ± 2.1 months is short. An extended observation period is necessary before making a final judgment.

In a previous report [32], the incidence of malignancy among our recipients was 3.9%. Kaposi sarcoma was the common type, and posttransplant lymphoproliferative disorder (PTLD) was the second most common one.

## 5. Conclusions

Factors affecting graft survival following renal transplantation from living donors were studied by univariate as well as multivariate analysis. Five variables were identified as factors with an independent impact on graft survival. Excellent outcomes were obtained at 5 years following transplantation. Thereafter, a steady decline was observed presumably as a result of chronic allograft nephropathy. Since the current immunosuppressive regimens play a central role in its pathogenesis, relentless efforts should be made to define the optimal steroid and/or calcineurin inhibitors sparing protocols.

## Figures and Tables

**Table:** (a)

	No. of patients	5-year survival %	95% CI		10-year survival %	95% CI		*P*-value (log rank)
Donor's sex								
Male	944	88.6	86.44	90.76	69.9	66.37	73.43	**0.012**
Female	1023	85.0	82.65	87.35	61.5	57.97	65.03	
Donor's age (years)								
≤30	772	87.7	85.35	90.05	68.5	64.58	72.42	**<0.001**
31–40	627	87.9	85.16	90.64	69.6	65.29	73.91	
41–50	389	83.4	79.48	87.32	58.0	52.12	63.88	
≥50	179	85.3	79.81	90.79	52.9	43.30	62.50	
Recipient's sex								
Male	1466	87.6	85.84	89.46	64.7	54.9	74.50	**0.829**
Female	501	84.3	80.97	87.63	67.8	63.10	72.50	
Recipient's age (years)								
≤20	378	84.4	80.68	88.12	59.0	52.92	65.08	**0.002**
21–30	709	88.6	86.25	90.95	66.9	62.78	71.02	
31–40	556	86.5	83.56	89.44	67.0	62.49	71.51	
41–50	270	85.7	81.39	90.01	67.4	60.54	74.26	
≥50	54	86.7	75.53	97.87	71.2	52.78	89.62	
The sex donor-recipient								
Male-male	693	89.5	87.15	91.85	68.2	63.89	72.51	**0.082**
Male-female	251	86.1	81.59	90.61	74.4	68.13	80.67	
Female-male	773	85.9	83.35	88.45	61.5	57.38	65.62	
Female-female	250	82.6	77.70	87.50	61.3	54.24	68.36	
Donor relationship								
Related	1620	87.3	85.54	89.06	65.7	62.96	68.44	**0.651**
Unrelated	347	84.3	80.18	88.42	64.6	58.33	70.87	

**Table:** (b)

	No. of patients	5-year survival %	95% CI		10-year survival %	95% CI		*P* value (log rank)
ABO compatibility								
Identical	1579	87.3	85.54	89.06	65.8	63.06	68.54	**0.500**
Different compatible	388	84.5	80.78	88.22	64.4	58.72	70.08	
Type of blood transfusion								
No transfusion	1049	89.1	87.14	89.80	69.0	65.28	72.72	**<0.001**
Third party	906	84.2	81.65	83.03	62.8	59.47	66.13	
Donor specific	12	—	—		—	—	
No. of blood transfusion								
No transfusion	1049	89.1	87.14	89.80	68.8	65.08	72.52	**0.001**
1-2 times	301	87.1	83.18	91.06	67.3	61.42	73.18	
3-4	265	82.1	77.40	86.80	64.4	58.13	70.67	
≥5	352	83.3	79.18	87.42	57.8	52.31	63.29	

**Table:** (c)

	No. of patients	5-year survival %	95% CI		10-year survival %	95% CI		*P* value (log rank)
HLA-A, B (class I)								
0-MM	153	93.2	89.08	97.32	71.4	62.38	80.42	**<0.001**
1-MM	229	92.6	89.07	96.13	74.1	67.44	80.76	
2-MM	996	86.2	84.04	88.36	62.7	59.17	66.23	
3-MM	309	82.5	77.99	87.01	62.4	56.13	68.67	
4-MM	132	85.7	79.43	91.97	71.0	62.18	79.82	
HLA-DR (class II)								
0-MM	201	91.5	87.58	95.42	77.6	69.56	85.64	**0.010**
1-MM	1720	86.7	84.94	88.46	64.5	61.76	67.24	
2-MM	2	—	—		—	—		
HLA (class I + II) MM								
0-MM	88	94.1	89.0	99.20	79.6	67.84	91.36	**0.001**
1-MM	102	93.8	89.09	98.50	68.0	57.02	78.98	
2-MM	234	90.0	86.08	93.92	71.3	64.64	77.96	
3-MM	968	86.2	83.85	88.55	62.8	59.08	66.52	
4-MM	290	82.4	77.89	86.91	62.0	55.53	68.47	
5-MM	131	85.6	79.33	91.87	69.3	60.28	78.32	
HLA-A, B, DR (class I and II) MM								
0/1/2	424	91.8	89.06	94.54	72.1	67.00	77.20	**0.011**
3/4/5/6	1389	85.8	83.44	87.36	63.4	60.46	66.34	
Genetic considerations								
HLA-ID sibling	152	94.4	92.64	96.16	79.2	70.18	88.22	**0.005**
1-Haplotype MM (R)	1322	87.7	85.94	89.46	64.7	61.56	67.84	
2-Haplotype MM (R + UR)	449	83.2	79.48	86.92	64.0	58.51	69.49	

**Table:** (d)

	No. of patients	5-year survival %	95% CI		10-year survival %	95% CI		*P* value (log rank)
Bilharziasis								
(i) Negative	1442	87.6	85.84	89.36	65.9	62.76	69.04	**0.796**
(ii) Positive	525	84.6	81.46	87.74	64.4	59.89	68.91	
Original kidney diseases								
(i) Glomerulonephritis	214	82.8	73.74	88.09	63.5	56.05	70.95	**0.020**
(ii) Chronic pyelonephritis	259	90.6	86.88	94.32	73.3	67.22	79.38	
(iii) Nephrosclerosis	50	84.9	74.51	95.29	55.4	39.72	71.08	
(iv) Obstructive uropathy	57	92.2	84.95	99.45	76.0	61.50	90.50	
(v) Amyloidosis	33	93.9	85.67	102.13	80.4	64.52	96.28	
(vi) Congenital polycystic	51	93.4	86.15	100.65	55.8	37.57	74.03	
(vii) Hypoplasia	17	100.0	100.00	100.00	83.1	61.54	104.66	
(viii) Others	126	80.9	73.65	88.15	58.8	45.67	71.93	
(ix) Not determined	1160	—	—	—	—	—	—	
Types of glomerulonephritis (GN)								
(i) Mesangiocapillary GN	35	81.8	68.67	94.93	62.7	42.90	82.50	**0.701**
(ii) Membranous GN	22	57.8	36.83	78.77	57.8	36.83	78.77	
(iii) F.S.G.S.	70	90.7	83.64	97.76	66.6	53.66	79.54	
(iv) Mesangioproliferative GN	23	73.9	55.87	91.93	67.8	53.66	79.54	
(v) Crescentic GN	16	87.5	71.23	103.77	46.7	17.69	75.71	
(vi) Hereditary nephritis	48	87.0	77.20	96.80	65.7	49.80	79.60	
Pretransplant hypertension								
(i) Normotensive	826	87.2	84.85	89.55	64.6	60.48	68.72	**0.466**
(ii) Hypertensive	1141	86.4	84.24	88.56	66.1	62.77	69.43	
No. of transplants received								
(i) First	1891	86.0	85.03	88.17	65.1	62.55	67.65	**0.242**
(ii) Second	73	90.0	82.94	97.06	77.8	67.22	88.38	
(iii) Third	3	—	—	—	—	—	

**Table 2:** Graft survival relative to technical variables.

	No. of patients	5-year survival %	95% CI		10-year survival %	95% CI		*P* value (log rank)
No. of renal arteries								
(i) Single	1749	86.8	85.23	88.37	66.0	63.26	68.74	**0.474**
(ii) Multiple	218	86.1	81.20	91.00	61.0	52.38	69.62	
Total ischemia time (min)								
<30	225	89.3	85.18	93.42	61.4	54.74	68.06	**0.039**
30–60	1506	86.2	84.44	87.96	65.7	62.76	68.64	
>60	236	87.8	83.29	92.31	74.2	66.56	81.84	
Time to diuresis								
(i) Immediate (<10 min)	1806	87.2	85.63	88.77	66.0	63.45	68.55	**0.029**
(ii) Delayed (>10 min)	161	81.5	75.03	87.97	59.2	49.99	68.41	
Primary urinary recontinuity								
(i) Ureterovesical (Lead better)	170	77.1	70.44	83.76	57.8	49.76	65.84	**0.624**
(ii) Ureterovesical (Leich Grieg)	1761	87.5	85.93	89.07	66.0	63.26	68.74	
(iii) Ureteroureteral	29	92.0	81.42	102.58	74.7	57.06	92.34	
(iv) Pyeloureteral	3	—	—	—	—	—	
(v) Ileal conduit	4	—	—	—	—	—	

**Table 3:** Graft survival relative to posttransplantation variables.

	No. of patients	5-year survival %	95% CI		10-year survival %	95% CI		*P* value (log rank)
Induction therapy								
No	1108	86.5	84.34	88.66	64.6	61.66	67.54	**0.536**
Yes	859	87.1	84.75	89.45	69.5	64.80	74.20	
Primary immunosuppression								
(i) Aza-based (dual therapy)	309	82.2	77.69	86.71	58.3	52.42	64.18	**<0.001**
(ii) CsA-based (dual therapy)	303	84.8	79.12	90.48	57.8	49.76	65.84	
(iii) CsA-based (triple therapy)	988	86.9	84.74	89.06	66.5	63.36	69.64	
(iv) Tacrolimus-based (triple therapy)	217	91.6	88.27	94.93	86.2	80.91	91.49	
(v) Sirolimus-based therapy	85	88.9	82.04	95.76	—	—	
Total steroid dose (during first 3 months)								
<5 gm	1158	89.3	87.34	91.26	70.3	66.77	73.83	**<0.001**
5–10 gm	601	85.0	82.06	87.94	64.8	60.68	68.92	
>10 gm	208	77.9	72.02	83.78	46.9	39.65	54.15	
No. of acute rejection episodes (during first 3 months)								
(i) No	708	94.2	92.44	95.96	82.1	78.18	86.02	**<0.001**
(ii) One	661	89.6	87.25	91.95	69.4	65.28	73.52	
(iii) ≥2	598	75.8	72.27	79.33	47.5	43.19	51.81	
Urological complications								
(i) No	1816	87.0	85.43	88.57	65.9	63.35	68.45	**0.449**
(ii) Yes	151	83.6	77.52	89.68	61.4	51.99	70.81	
Post-transplant hypertension								
(i) Normotensive	758	86.2	83.46	88.94	75.7	71.58	79.82	**<0.001**
(ii) Sustained hypertension	809	88.0	85.65	90.45	65.9	62.18	69.62	
(iii) Newly developed hypertension	400	85.7	82.17	89.23	56.0	50.71	61.29	

**Table 4:** Cox proportional hazard analysis.

Characteristic	Regression estimate (*B*)	S.E.	Relative risk Exp(*B*) (95% CI)	*P* value
Donor's age (years)				
<30	—	—	1	
31–40	−0.049	0.097	0.952 (0.788, 1.150)	** 0.609**
41–50	0.326	0.109	1.385 (1.120, 1.714)	** 0.003**
>50	0.487	0.145	1.628 (1.224, 2.164)	**<0.001**
Genetic considerations				
(i) HLA-ID siblings	—	—	1	
(ii) 1-haplotype MM (R)	0.059	0.203	1.099 (0.739, 1.635)	** 0.641**
(iii) 2-haplotype MM (R + UR)	0.404	0.211	1.498 (0.990, 2.267)	**0.056**
No. of acute rejection episodes (during first 3 months)				
(i) No	—	—	1	
(ii) One	0.779	0.130	2.218 (1.689, 2.814)	**<0.001**
(iii) ≥2	1.559	0.131	4.754 (3.737, 6.141)	**<0.001**
Total steroid dose (during first 3 months)				
<5 gm	—	—	1	
5–10 gm	−0.363	0.100	0.696 (0.572, 0.846)	**0.001**
>10 gm	−0.294	0.135	0.745 (0.572, 0.972)	**0.030**
Primary immunosuppression				
(i) Aza-based (dual therapy)	—	—	1	
(ii) CsA-based (dual therapy)	−0.022	0.139	0.978 (0.744, 1.285)	**0.872**
(iii) CsA-based (triple therapy)	−0.234	0.114	0.791 (0.633, 0.989)	**0.039**
(iv) Tacrolimus-based (triple therapy)	−0.686	0.228	0.504 (0.322, 0.787)	**0.003**
